# Reciprocal regulation of *PCGEM1* and *miR-145* promote proliferation of LNCaP prostate cancer cells

**DOI:** 10.1186/s13046-014-0072-y

**Published:** 2014-09-10

**Authors:** Jin-Hua He, Jing-zhi Zhang, Ze-Ping Han, Li Wang, Yu Bing Lv, Yu-Guang Li

**Affiliations:** Department of Laboratory, Central Hospital of Panyu District, 8 Fuyu Dong Road, shiqiao, Guangzhou, Guangdong 511400 P R China; The Second Affiliated Hospital of Guangzhou Medical University, Guangzhou, 510620 China

**Keywords:** Long non-coding RNA, MicroRNA-145, Prostate cancer gene expression marker 1, Prostate cancer cells, Small interfering RNA sequences, Reciprocal regulation

## Abstract

Prostate cancer gene expression marker 1 (*PCGEM1*) is a long non-coding RNA (lncRNA) overexpressed in prostate cancer (PCa) cells that promotes PCa initiation and progression, and protects against chemotherapy-induced apoptosis. The microRNA *miR-145* functions as a tumor suppressor in PCa. We speculate that reciprocal regulation of *PCGEM1* and *miR-145 promote proliferation of LNCaP prostate cancer cells. To test this hypothesis,* the interaction between *PCGEM1 *and *miR-145* was examined using a luciferase reporter assay. Expression levels *w*ere selectively altered in LNCaP cells and noncancerous RWPE-1 prostate cells by transfection of *miR-145* or small interfering RNA sequences against (siRNA) *PCGEM1*. Relative expression levels were detected by RT-PCR, tumor cell growth and early apoptosis by the MTT assay and flow cytometry, respectively, and tumor cell migration and invasion properties by transwell assays. The effect of siRNA *PCGEM1* and *miR-145* transfection on prostate cancer growth in vivo was examined in the (nu/nu) mouse model. *PCGEM1* and *miR-145* exhibited reciprocal regulation; downregulation of PCGEM1 expression in LNCaP cells increased expression of *miR-145*, while overexpression of *miR-145* decreased *PCGEM1 *expression. Transfection of the *miR-145* expression vector and siRNA *PCGEM1* inhibited tumor cell proliferation, migration, and invasion, and induced early apoptosis both in vitro. In contrast, there was no effect on RWPE-1 cells. We demonstrate a reciprocal negative control relationship between *PCGEM1* and *miR-145* that regulates both LNCaP cell proliferation and nu/nu PCa tumor growth. The results also identify *PCGEM1* and associated regulators as possible targets for PCa therapy.

## Background

Long non-coding RNAs (lncRNAs) are untranslated transcripts longer than 200 nucleotides baring many of the structural characteristics of mRNAs, including a polyA tail, 5′-capping, and a promoter structure, but no conserved open reading frame [1-6]. Many lncRNAs are expressed at specific times and in specific tissues during development, and exhibit a variety of slicing patterns. It has been proposed that lncRNAs are involved in the epigenetic regulation of coding genes, and thus exert a powerful effect on a number of physiological and pathological processes, including the pathogenesis of many human cancers [7-11].

MicroRNAs (miRs) are small noncoding RNAs usually 20–22 nucleotides long. To date, close to 1000 human miRs have been identified. Collectively, miRs are thought to regulate more than 50% of all human genes by binding to mRNA sequences and repressing expression, either by inhibiting translation or promoting RNA degradation [12-17].

Given the structural similarly with mRNAs, lncRNAs may be another important member of the non-coding RNA family [18]. The interaction between lncRNAs and miRs has been linked to the invasion and metastasis of tumors [19]. For example, the *miR-29a* epigenetically modulated expression of the lncRNA *MEG3* in hepatocellular carcinoma (HCC) through promoter hypermethylation [20]. Loss of miR-31 expression in triple-negative breast cancer (TNBC) lines is attributed to hypermethylation of its promoter-associated CpG islan. *MicroRNA-31* anchors the novel lncRNA *LOC554202* and adjusts its transcriptional activity [21]. Moreover, the lncRNA *HULC* can inhibit the expression of the tumorigenic *miR-372* [22].

Prostate cancer gene expression marker 1 (*PCGEM1*) is part of a novel class of androgen-regulated lncRNAs [23]. Overexpression in prostate cancer (PCa)-derived LNCaP cells promotes proliferation and a dramatic increase in colony formation [24,25]. Many miRs function as oncogenes or tumor suppressors in human cancers [26-32]. Downregulation of miR-145 has been reported in PCa, suggesting that *miR-145* functions as a tumor suppressor [33]. Using the biology information software RegRNA (http://regrna.mbc.nctu.edu.tw/), we predicted that 48 distinct miRs bind to *PCGEM1*. Further online comprehensive analysis (http://cbio.mskcc.org/cancergenomics/prostate/data/) indicates that 96 miRs are associated with PCa. Clustering intersection analysis also linked *miR-145* with PCa. Significantly, *miR-145* has a binding site for lncRNA; thus, reciprocal regulation of *PCGEM1* and *miR-145* may promote or suppress PCa cell proliferation [34]. In this study, we explored possible mutual regulation of *PCGEM1* and *miR-145* expression in prostate cancer and the impact on PCa cell proliferation and invasive capacity.

## Materials and methods

### Materials

Non-cancerous RWPE-1 cells, HEK293T cells and LNCaP cells were purchased from the Shanghai Institute of Cell Biology (Shanghai, China). RPMI 1640 medium, fetal bovine serum (FBS), and Lipofectamine 2000 were obtained from Invitrogen (Carlsbad, CA, USA). The restriction enzymes NotI and XhoI, T4 DNA ligase, and reagents for RT-PCR were purchased from TaKaRa (Takara BioInc, Shiga, Japan). 3-(4,5-Dimethylthiazol-2-yl)-2,5-diphenyltetrazolium bromide (MTT), annexin-V-FITC, and propidium iodide (PI) were purchased from Sigma Chemical (USA), and negative control sequences and negative control inhibitor sequences were purchased from Ruibo Company (Shanghai, China).

### Design and construction of eukaryotic expression vector for hsa-miR-145

The mature hsa-miR-145 sequence (5′-GUCCAGUUUCCCAGGAAUCCCU-3′) is available from the miRNA Registry (MIMATOOOO437). To prevent formation of a termination signal, TTGGCCACTGACT was selected as the region in a miR expression vector template. The sequence TGCT was added to the 5′ positive-sense strand template of the miR expression vector and GTCC to the 5′ antisense strand template. Further, a nonspecific sequence was designed and sent to Shanghai GenePharma Co, Ltd. for synthesis. The assay was according to previously described [35]. The eukaryotic expression vector plasmid targeting hsa-miR-145 was named *pmiR-145*.

### Design and synthesis of siRNA

siRNAs are methylated 21 bp double-stranded RNA oligonucleotides. It uses gene-specific targets for RNAi analysis and reports up to 10 top scoring siRNA targets. The freeze-dried siRNAs were dissolved in RNase-free water and stored as aliquots at −20°C. The siRNA sequence of PCGEM1 (sense: 5′-GCCCUACCUAUGAUUUCAUAU-3′, antisense: 5′-AUAUGAAAUCAUAGGUAGGGC-3′) and negative control sequence (sense: 5′-UUCUCCGAACGUGUCACGUUUC-3′ antisense: 5′-GAAACGUGACACGUUCGGAGAA-3′) were synthesized by Shanghai GenePharma (Shanghai, China).

### Grouping and cell transfection

The experimental culture groups included 1) untransfected LNCaP and RWPE-1 cells (control groups), 2) cells transfected with *pmiR-145* or *miR-145* mimics (1.6 μg/ml and 50 nM, respectively), 3) cells transfected with the scrambled nucleotide sequence and empty vector (negative control or NC groups, 50 nM), 4) cells transfected with a miRNA inhibitor (NI group, 100 nM), 5) a negative control for NI (NCI group, 50 nM), 6) cells transfected with siRNA *PCGEM1* sequence (siRNA *PCGEM1* group, 50 nM). Cells in log phase growth were seeded on 6-well culture plates (2 × 10^5^ cells/well) and transfected when the cell fusion rate reached 70%. The DNA Lipofectamine 2000 or RNA Lipofectamine 2000 compound was added according to the manufacturer’s instructions (Invitrogen). After 6 h, the transfection medium was discarded. Cells were washed with serum-free RPMI 1640 and then cultured in RPMI 1640 supplemented with 10% FBS.

### Luciferase reporter assay

The whole mRNA sequences of the *PCGEM1* gene were obtained by PCR amplification and cloned separately into multiple cloning sites of the psi-CHECKTM-2 luciferase miRNA expression reporter vector. HEK293T cells were transfected with miR-145 mimic, miR-145 inhibitor, a control miRNA, a miRNA inhibitor control, or empty plasmid using Lipofectamine 2000 according to the manufacturer’s instructions. Nucleotide-substitution mutation analysis was carried out using direct oligomer synthesis of PCGEM1 sequences. All constructs were verified by sequencing. Luciferase activity was measured using the dual luciferase reporter assay system kit (Promega Co, Madison, WI, USA) according to the manufacturer’s instructions on a Tecan M200 luminescence reader.

### Quantitative real-time RT-PCR

Total RNA samples were extracted using Trizol (Invitrogen, CA) according to the manufacturer’s instructions. Real-time quantitative PCR analysis was performed using an Applied Biosystems 7500 Real-Time PCR Systems (Applied Biosystems, Foster City, CA). The expression level of *18S* was used as an internal control for mRNAs, and *U6* level as an internal control for miRNAs. Primers used in quantitative real-time PCR analysis were: *U6* (forward: 5′-CTCGCTTCGGCAGCACA -3′, reverse: 5′- AACGCTTCACGAATTTGCGT-3′); *18S* (forward: 5′-CCTGGATACCGCAGCTAGGA-3′, reverse: 5′-GCGGCGCAATACGAATGCCCC-3′); *miR-145* (RT primer: 5′-CTCAACTGGTGTCGTGGAGTCGGCAATTCAGTTGAGTTCCCAT-3′, forward: 5′-ACACTCCAGCTGGGGTCCAGTTTTCCCAGGAA-3′, reverse: 5′-CTCAACTGGTGTCGTGGA-3′); *PCGEM1* (forward: 5′-CACGTGGAGGACTAAGGGTA-3′, reverse: 5′-TTGCAACAAGGGCATTTCAG-3′); The expression level was calculated using CT and 2^-ΔΔCt^.

### MTT assay

The viability of LNCaP and RWPE-1 cells was determined by MTT assay. Briefly, cells at 5 × 10^4^/ml were transfected with siRNA *PCGEM1* (siRNA *PCGEM1* groups, 50 nM), empty plasmid and scramble sequence (negative control group, 1.6 μg/ml), or pmiR-145 (pmiR-145 group, 1.6 μg/ml) in the presence of Lipofectamine 2000 and serum-free RPMI 1640 media for 6 h. Cells were plated in 96-well plates in medium containing 10% FBS for another 24, 48, or 72 h. MTT stock solution (20 μl, 5 mg ⁄ml) was added to each well for a final MTT concentration of 0.45 mg⁄ml and the plate was incubated for 4 h at 37°C. Media was then removed and dimethylsulfoxide (DMSO) (150 μl added to dissolve the blue formazan crystals (the product of MTT conversion by viable cells) at room temperature for 30 min. The relative change in viable cell number was estimated by absorbance at 570 nm on a Bio-Rad microtiter plate reader (Hercules, CA, USA).

### Flow cytometry assay

LNCaP and RWPE-1 cells were seeded at 1.0 × 10^6^/ml in 24-well plates (Costar) and transfected in 500 μl media/well siRNA *PCGEM1* (siRNA *PCGEM1* groups, 50 nM), empty plasmid and scramble sequence (negative control group, 1.6 μg/ml), or pmiR-145 (pmiR-145 group, 1.6 μg/ml) by Lipofectamine 2000 reagent (Invitrogen) in serum-free RPMI 1640 for 6 h. After transfection, 500 μl of the appropriate growth medium containing 20% FBS were added to each well. Cells were incubated for another 48 h then harvested, washed twice with PBS, fixed with 70% ethanol, and treated with RNase A (1 mg/ml). Finally, the cells were double-stained with FITC-conjugated annexin-V and propidium iodide (PI) solution (50 μg/ml). For each sample, data from approximately 10000 cells were recorded in the list mode on logarithmic scales. Apoptosis and necrosis were analyzed by quadrant statistics on double negative, annexin-V-positive/PI-negative, annexin-V-negative/PI-positive, and double-positive cells.

### Migration and invasion assay

Cells were transfected with siRNA *PCGEM1* (siRNA *PCGEM1* groups, 50 nM), empty plasmid and scramble sequence (negative control group, 1.6 μg/ml), or pmiR-145 (pmiR-145 group, 1.6 μg/ml) by Lipofectamine 2000 reagent in serum-free RPMI 1640 for 6 h. One day after transfection, 1 × 10^5^ cells were collected, resuspend in 100 μl basal medium, and transferred to the transwell chamber. A 600 μl volume of complete medium was added to the well and the chamber inserted. Plates were incubated at 37°C for 48 h. Remaining cells were swabbed from the top transwell membrane filter and the chamber submerged in 4% paraformaldehyde for 20 min. Cells in the well were stained with crystal violet for 10 min, washed in PBS buffer, then counted by light microscope to determine transwell migration.

The transwell assay was performed as above except with Matrigel in the wells of 24-well plates. The Matrigel was first incubated in pre-chilled basal medium (40 μl) at 37°C for 2 h. The excess medium was discarded, 100 μl basal medium added to the well with the Matrigel and 600 μl to the chamber, following by incubation at 37°C overnight. Transfected cells (1 × 10^5^) were resuspend in 100 μl basal medium and transfer to the transwell chamber. Then, 600 μl complete medium was added to the well and the chamber inserted. The plates were incubated at 37°C for 24 or 48 h. Cells were stained with crystal violet for 10 min, washed with the PBS and counted using an inverted microscope.

### In vivo treatment

BalB/c (nu/nu) mice from the Animal Center of Guangzhou Province (Guangdong, China) received subcutaneous injections of 2 × 10^6^ LNCaP cells into each axilla area. When xenograft tumors became palpable (about 0.1 mm^3^), mice were randomly divided into the control group receiving PBS injection (100 μl), siRNA *PCGEM1* (500 nM), negative control (plasmid, scramble sequence 16 μg), PmiR-145 (16 μg), with 6 mice each group. There was no difference in baseline tumor size between the groups. Tumor volume was calculated every 3 days according to the formula v = ab2π/6, where “a” is the maximum tumor diameter and “b” the minimum diameter. After treatment for 20 d, mice were euthanized and tumors were dissected and weighed.

### Data analysis

All results are the averages of at least three independent experiments from separately treated and transfected cultures. Data are expressed as the mean ± SD. Statistical comparisons were made by one-way analysis of variance (ANOVA). P < 0.05 was considered to indicate a statistically significant difference.

## Results

### miR-145 regulates *PCGEM1* expression by binding to the *PCGEM1*

**Figure 1:**
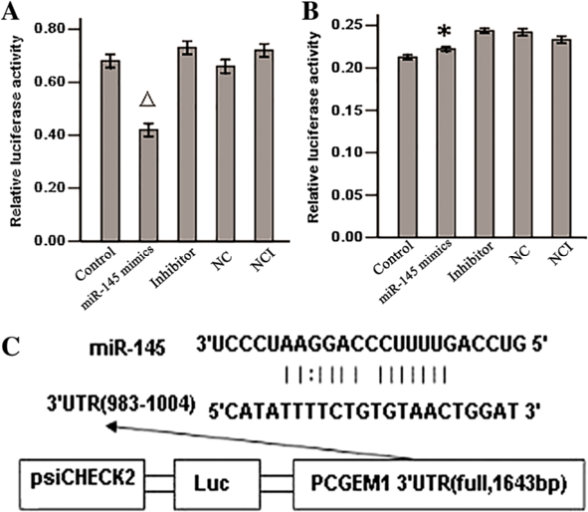
**The interaction between** ***miR-145*** **and** ***PCGEM1*** **was detected by sensor reporter. A**: Comparison of luciferase activity of plasmid transfected cloned *PCGEM1* (P^△^ < 0.05) vs control and NC. **B**: The luciferase activity comparison of empty psiCHECK-2 (P^*^ > 0.05) vs control and NC, IN, NC, NCI group. **C**: The target sequence for *miR-145* at 1: Control group; 2: *miR-145* mimics group; 3: inhibitor group; 4: NC group; 5: NCI group.

ReRNA, an lncRNA target miR prediction software, predicted miR-145 binding sites at 983 bp and 1004 bp in the *PCGEM1* (Figure 1C). The full-length sequence of *PCGEM1* (1643 bp) was cloned downstream of the luciferase gene in the psiCHECK carrier to construct the psiCHECK-2-*PCGEM1* carrier. Co-transfection of HEK293T cells with miR-145 mimics was performed to detect the binding site on the *PCGEM1* (983–1004 bp). The co-transfection of HEK293T cells with miR-145 mimics and psiCHECK-2-*PCGEM1* also significantly inhibited luciferase activity (*P* <0.05) (Figure 1A); however, co-transfection of HEK293T cells with miR-145 mimics and empty psiCHECK-2 had little effect on the activity of luciferase (*P* > 0.05) (Figure 1B). We concluded that miR-145 can regulate the expression of *PCGEM1* by directly binding to target sites within the *PCGEM1* sequence.

### The *miR-145* and *PCGEM1* expression in in the LNCaP cells exhibited reciprocal regulation

**Figure 2:**
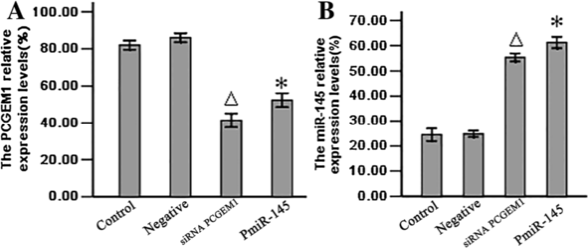
**The relative expression levels of** ***PCGEM1*** **and** ***miR-145.*** **A**: The *PCGEM1* mRNA expression levels were determined by Real time RT-PCR, P^△*^ < 0.05 vs control and NC group. **B**: The *miR-145* expression levels determined by Real time RT-PCR, P^△*^ < 0.05 vs control and NC group. 1: Control group; 2: Negative group: 3: siRNA *PCGEM1* group. 4. *PmiR-145* group.

To examine possible mutual regulation of *PCGEM1* and *miR-145*, we designed a small interfering RNA sequences to knockdown *PCGEM1* expression and detected the expression levels of miR-145. Alternatively, we transfected *PmiR-145* into LNCaP cells and then detected the expression levels of *PCGEM1*. Transfection of the siRNA *PCGEM1* downregulated expression of *PCGEM1* and resulted in a significant increase in *miR-145* expression compared to the NC group and control group (both P < 0.05) (Figure 2).

### siRNA-mediated *PCGEM1* knockdown and *miR-145* overexpression inhibited LNCaP proliferation

**Figure 3:**
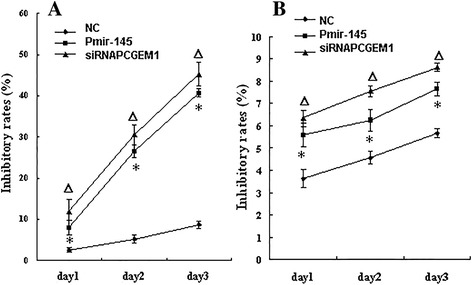
**The viability of cells determind by MTT assay.** LNCaP cells after transfection, the cells were plated in 96-well plates in medium containing 10% FBS for another 24, 48, 72 hour. Cell viability was assessed by MTT assays. Mean values of three independent experiments with standard errors are represented. **A**: The inhibitory rates of LNCaP cells, P△* < 0.05 vs NC group. **B**: The inhibitory rates of RWPE-1cells, P△* > 0.05 vs NC group.

To examined the effect of siRNA-mediated *PCGEM1* knockdown and concomitant overexpression of miR-145 on PCa cell proliferation, normal prostate cells (RWPE–1) and LNCaP cells were transfected as above and cell numbers estimated by MTT after 24, 48, 72 h. Indeed, both siRNA *PCGEM1* and *PmiR-145* transfection groups exhibited a significant reduction in LNCaP cell proliferation, while transfection with empty vector and scrambled sequences (negative control group) had no effect compared to untransfected controls (Figure 3A). In contrast, proliferation of RWPE-1 cell was not changed significantly, even after 72 h (Figure 3B).

### siRNA-mediated *PCGEM1* knockdown and *miR-145* overexpression induced early apoptosis of LNCaP cell

**Figure 4:**
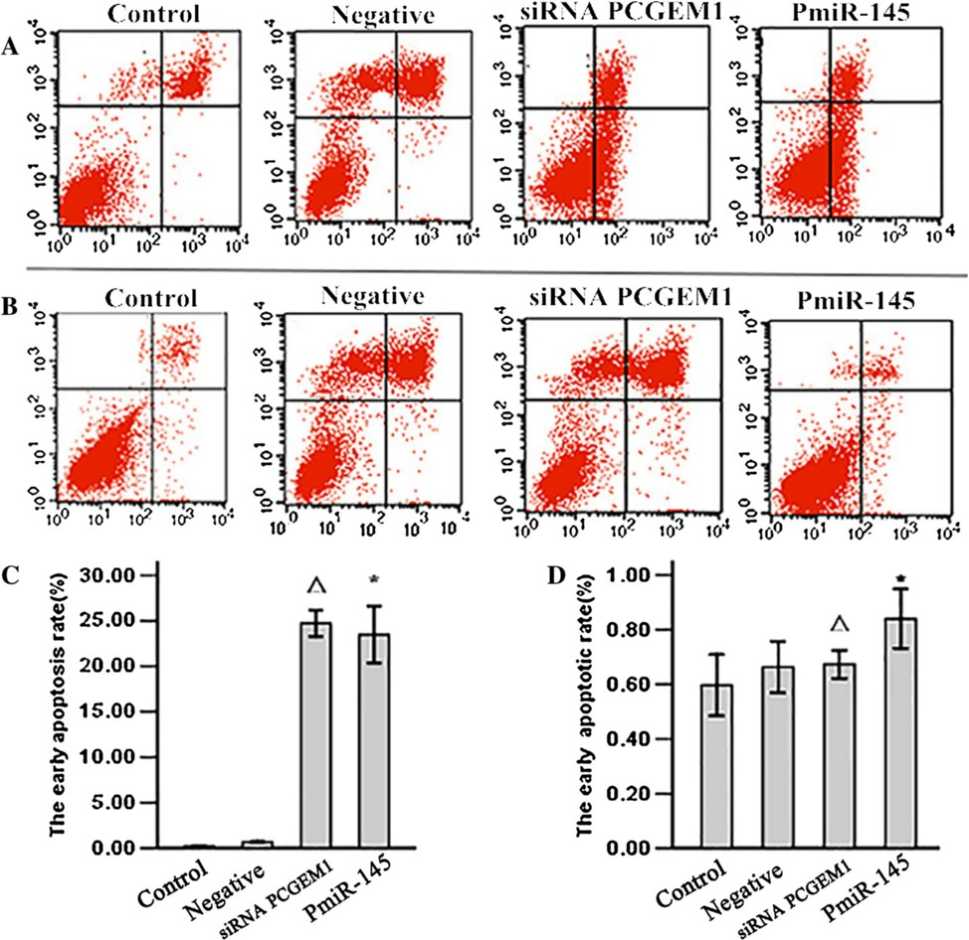
**The cell early apoptotis rate was detecte by flow cytometry.** LNCaP and RWPE-1 cells cells were serum-starved for 6 h and then transfected with siRNA *PCGEM1 *or *PmiR-145*. After 48 h cells were collected and fixed in paraformaldehyde and ethanol overnight. Cells were stained by propidium iodide (PI) and with FITC-conjugated AnnexinV, analyzed by flow cytometry with a BD FACScalibur (BD Bioscience, Heidelberg, Germany). Mean values of three independent experiments with standard errors are represented. **A**: Representative Annexin V/PI double staining LNCaP cells. **B**: Representative Annexin V/PI double staining RWPE-1 cells. **C**: Comparison of the percentage of early apoptosis in LNCaP cells, P^△*^ < 0.05 vs control and NC group. **D**: Comparison of the percentage of early apoptosis in RWPE-1 cells, P^△*^ > 0.05 vs control and NC group. 1: Control group; 2: Negative group: 3: siRNA *PCGEM1* group. 4. *PmiR-145* group.

Cell apoptosis of the above transfection groups was detected by flow cytometry using annexin-V/PI double staining. In total, 25.16% of *PCGEM1* knockdown and 23.6% of miR-145-overexpressing LNCaP cells were in early apoptosis (Figure 4A, C), compared to only 0.63% and 0.82% of RWPE-1 cells, respectively, in these same transfection groups (Figure 4B, D).

### siRNA-mediated *PCGEM1* knockdown and *miR-145* overexpression decreased LNCaP cell migration and invasion

**Figure 5:**
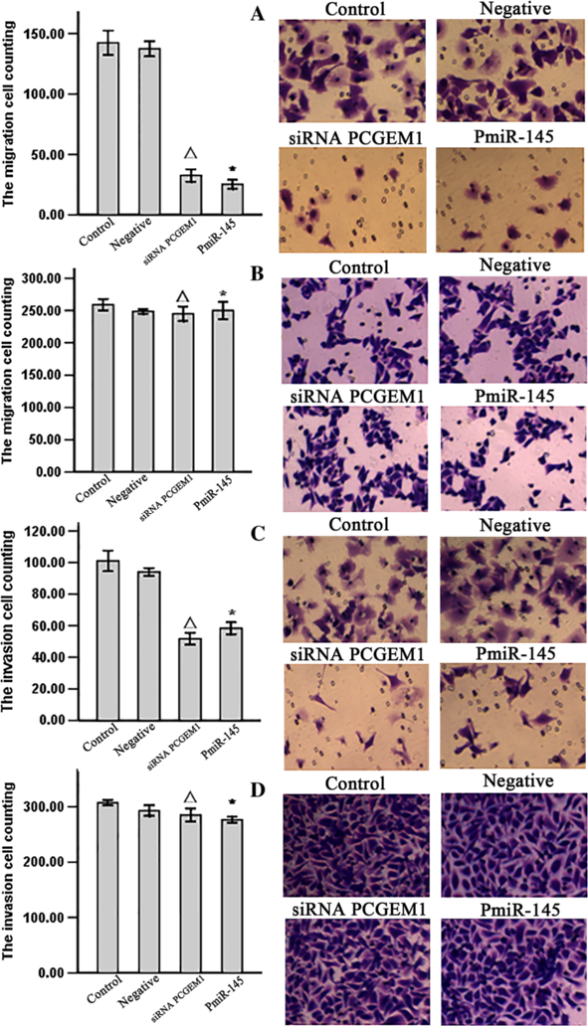
**Effect of siRNA** ***PCGEM1/PmiR-145*** **on cell migration.** After 48 h, migrated and invasion cells were fixed, stained, and counted. siRNA *PCGEM1* and *PmiR-145* significantly decreased LNCaP cell migration and invasion and have no effcets on RWPE-1 cell migration. **A**: a Transwell migration assay was done on LNCaP cells with siRNA *PCGEM1* and *PmiR-145*. P^△^* < 0.05 vs control and NC group. **B**: a Transwell migration assay was done on LNCaP cells with siRNA *PCGEM1* and *PmiR-145*. P^△^* > 0.05 vs control and NC group. **C**: a Transwell invasion assay was done on LNCaP cells with siRNA *PCGEM1* and *PmiR-145*. P^△^* < 0.05 vs control and NC group. **D**: a Transwell invasion assay was done on LNCaP cells with siRNA *PCGEM1* and *PmiR-145*. P^△^* > 0.05 vs control and NC group 1: Control group; 2: Negative group: 3: siRNA *PCGEM1* group. 4. *PmiR-145* group.

Transwell migration assays revealed that *PCGEM1* knockdown led to a mean 46 ± 8.6% decrease in LNCaP cell migration, and miR-145 overexpression to a mean 36 ± 7.8% decrease (Figure 5A), while these treatments had no effect on REWP-1 cell migration (Figure 5B). The effect of siRNA *PCGEM1*/*PmiR-145* on LNCaP cell invasion was evaluated using Matrigel-coated Transwell invasion assays. siRNA *PCGEM1* led to a mean 52 ± 6.6% decrease in LNCaP cell invasion and pmiR-145 led to a mean 59 ± 6.3% decrease in LNCaP cell invasion (Figure 5C). siRNA *PCGEM1* and *PmiR-145* have no effects on RWPE-1 cell invasion (Figure 5D).

### siRNA-mediated *PCGEM1* knockdown and *miR-145* overexpression inhibits progression of tumor xenografts

**Figure 6:**
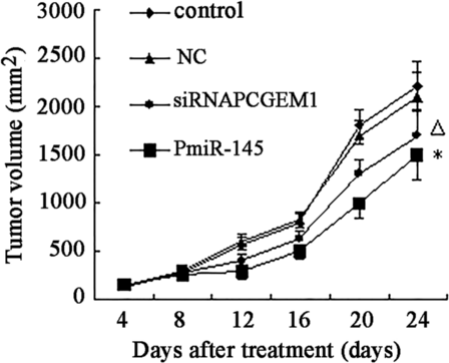
**Effect of siRNA** ***PCGEM1*** **/** ***pmiR-145*** **on prostate cancer xenografts.** LNCaP tumor xenografts were established in male Athymic nude mice. Animals in the treatment arm received siRNA *PCGEM1* (500 nM /kg once daily) or *pmiR-145* (16 μg/kg once daily). Control mice received PBS (100 μl/kg once daily). Negative Control mice received empty plasmid or scrambled sequence (16 μg/kg once daily). After 20 daily treatments, tumors injected with siRNA *PCGEM1* or *pmiR-145* were significant smaller than control or NC group tumors (P^△^* < 0.05).

LNCaP tumor xenografts were established in Athymic nude mice to evaluate the effects of siRNA* PCGEM1* or *pmiR-145* on prostate cancer growth in vivo. Compared to the untreated animals, application of siRNA *PCGEM1* or *pmiR-145* significantly diminished the tumor volume, whereas negtive control group had no effect (Figure 6). No body weight loss or diarrhea was observed and all animals (treated as well as non-treated) survived. The results shows that shows that reduce the expression of *PCGEM1* or over express *miR-145* can effectively inhibit prostate cancer growth in vivo.

## Discussion

Long non-coding RNAs (lncRNAs) are a new class of regulatory RNA [36]. These mRNA-like molecules, which lack significant protein-coding capacity, were once thought to be a part of the genomic “dark matter”, but recent studies have implicated lncRNAs in a wide range of biological functions through poorly understood molecular mechanisms [37]. Despite recent insights into how lncRNAs function in such diverse cellular processes as regulation of gene expression and assembly of cellular structures, by and large, the key questions regarding lncRNA mechanisms remain to be answered [38]. The lncRNA Prostate cancer gene expression marker 1 (*PCGEM1*) is overexpressed in PCa, suggesting roles in proliferation, metastasis, and invasion [39]. In order to reveal the mechanisms regulating expression in PCa, we have predicted PCGEM1 interaction with miR-145 using billogical information (Figure 1C), and futher investigated a possible interaction with the tumor suppressor *miR-145.* Co-transfection of LNCaP cells with *miR-145* mimics or miR-145 inhibitor with psiCHECK-2-*PCGEM1* significantly inhibited reporter gene activity but only miR-145 suppressed reported gene expression when transfected with empty psiCHECK-2 (Figure 1A, B). Thus, *miR-145* may regulate *PCGEM1* expression by directly binding to target sites within the *PCGEM1* sequence.

We then demonstrated a mutual inhibitory control relationship between *PCGEM1* and *miR-145* by selective siRNA-mediated *PCGEM1* knockdown and miR-145 overexpression. Expression of *PCGEM1* (locus 2q32) was detected in the androgen receptor-positive cell line LNCaP but not in noncancerous prostate lines or androgen-receptor negative Pca lines [23]. *PCGEM1* overexpression in LNCaP cells promotes cell proliferation and a dramatic increase in colony formation, suggesting a role in cell growth regulation [24]. In contrast, miR-145 expression was low in all the prostate cell lines tested (PC3, LNCaP, and DU145) compared to the normal cell line RWPE-1, and in cancerous regions of human prostate tissue compared to adjacent normal prostate tissue [39]. To test the possibility of mutual negative regulation of *PCGEM1* and *miR-145,* we design a small interfering RNA targeting *PCGEM1* and a vector for *miR-145* overexpression (*pmiR-145*) and transfected these into LNCaP cells and normal RWEP–1 cells. RT-PCR results showed that knockdown of *PCGEM1* in LNCaP cells increased *miR-145* expression (Figure 2A) and that miR-145 overexpression reduced *PCGEM1* expression (Figure 2B). Inhibition of *PCGEM1* reduced LNCaP proliferation (Figure 3A), transwell migration and invasive capacity into Matrigel (Figures 5A, 6A), and the growth of solid tumors, possibly by promoted early apoptosis (Figure 4A). However, altering *PCGEM1* expression had no significant effect on RWPE-1 cell growth, migration, or invasion (Figures 3, 4, 5, 6B). The proliferation, colony formation, and soft agar growth of liver cancer cells was reduced by inhibiting expression of the lncRNA TUC339 using an siRNA [40], while silencing HULC expression in hepatoma effectively inhibited the growth of liver cancer cells [41]. In contrast, siRNA gene silencing of *MEG3* expression promote cell proliferation, whereas overexpression inhibited proliferation and promoted apoptosis [42]. Thus, individual lncRNAs can either promote or inhibit carcinogenesis. Selective knockdown and overexpression of lncRNAs may be feasible strategies to reduce tumor growth in a variety of tissue. Specifically, *miR-145* is a well documented tumor suppressor [43-46], and we successfully constructed an overexpressing vector that suppressed PCa cell growth with no observable effects on noncancerous prostate cells. Similarly, gain-of-function assays revealed that *miR-145* transfection inhibited cell proliferation, migration and invasion of PC3 and DU145 PCa cell lines [47].

While *PCGEM1* is known to be overexpressed in PCa, it is unknown if overexpression directly causes hyperproliferation and (or) metastasis [24]. Fu [48] found that overexpression of *PCGEM1* attenuated doxorubicin-induced expression of *p53* and *p21Waf1/Cip*1, and inhibited apoptosis of LNCaP cells. Petrovics [24] revealed that elevated *PCGEM1* expression increased cell proliferation and Rb phosphorylation. However, to the best of our knowledge, no study has investigated *PCGEM1* regulation by *miR-145*. An siRNA *PCGEM1* inhibited LNCaP cells growth and reduced migration and invasion, likely by raising the expression levels of *miR-145*.

Indeed, we confirmed direct binding of *miR-145* to the *PCGEM1* and demonstrated reciprocal regulation of these two transcripts. Moreover, *miR-145*-mediated suppression of *PCGEM1* suppressed tumor growth in vivo and PCa cell proliferation and invasive capacity in vitro. In turn, Reciprocal regulation of *PCGEM1* and *miR-145* promote proliferation of LNCaP prostate cancer cells.

In conclusion, our study demonstrates reciprocal negative control of *PCGEM1,* a tumor-promoting long noncoding RNA, and the tumor suppressor *miR-145.* This study highlights the interrelationship between two classes of non-coding RNAs. Both downregulation of *PCGEM1* or overexpression of the *miR-145* reduced the proliferation and invasive capacity of prostate cancer cells in vitro and in vivo.

## Abbreviations

lncRNA: Long non-coding RNA
PCGEM1: Prostate cancer gene expression marker 1
miRs: microRNAs
siRNA: Small interfere RNA
MTT: 3-(4,5-dimethylthiazol-2-yl)-2,4-diphenyl-tetrazo-lium bromide
FBS: Fetal bovine serum
DMSO: Dimethyl sulfoxide
PI: Propidium iodide
